# Association between air pollution and cerebrospinal fluid alpha-synuclein in urban elders: the CABLE study

**DOI:** 10.3389/fnagi.2024.1422772

**Published:** 2024-08-30

**Authors:** An-Yi Wang, He-Ying Hu, Yan Sun, Ya-Nan Ou, Ya-Hui Ma, Meng Li, Qiong-Yao Li, Lan Tan

**Affiliations:** Department of Neurology, Qingdao Municipal Hospital, Qingdao University, Qingdao, China

**Keywords:** air pollution, alpha-synuclein, cerebrospinal fluid, synucleinopathiess, association

## Abstract

**Introduction:**

Increasing evidence suggests that air pollution has a significant impact on the development of synucleinopathies, but the potential neurobiological mechanisms are unknown. We aimed to explore the associations of air pollution (including ozone [O_3_], nitrogen dioxide [NO_2_], and particulate matter [PM_2.5_]) with CSF α-syn levels in urban older adults.

**Methods:**

We included 933 urban participants from the Chinese Alzheimer’s Biomarker and LifestylE study. The 5-year average levels of air pollution exposure were estimated in the areas of residence. Multivariate linear regression was conducted to detect the correlation of air pollution with CSF α-syn levels. Subgroup analyses by age, gender, season, and history of coronary heart disease (CHD) were performed. Moreover, restricted cubic spline (RCS) models were applied to explore the potential nonlinear relationships.

**Results:**

We found a significant correlation of CSF α-syn level with PM_2.5_ in urban participants. Specifically, multiple linear regression showed a significant negative association between PM_2.5_ and CSF α-syn level (*p* = 0.029), which was more significant in female, midlife, non-CHD, and cold season subgroups. Besides, RCS models showed that O_3_ had an inverse J-shaped association with CSF α-syn levels in urban participants (*p* for nonlinearity = 0.040), and the harmful effect possibly appeared when O_3_ was above 37.9 ppb.

**Discussion:**

Long-term exposure to air pollution was associated with lower CSF α-syn levels, which may offer a new direction for exploring and preventing synucleinopathies.

## Background

Concerns are growing about the negative impacts of air pollution on human health since the twentieth century (Fowler et al., 2020). According to the 2016 World Health Organization estimation (World Health Organization, 2018), air pollution was responsible for approximately 4.2 million premature deaths. Recently, substantial evidence has suggested that air pollution may harm the brain and contribute to many neurodegenerative diseases (Costa et al., 2020). Since currently there is no cure for these neurodegenerative diseases, early prevention and intervention of their risk factors are critical.

Alpha-synuclein (α-syn), a neuronal protein abundant in synaptic terminals, has been considered to play a vital role in neuronal degeneration. These neurodegenerative diseases characterized by misfolded oligomers and aggregation of α-syn are collectively referred to as synucleinopathies, which include Parkinson’s disease (PD), multiple system atrophy (MSA), and diffuse Lewy Body Disease (DLB) (Burré et al., 2018). Compared to blood and saliva, cerebrospinal fluid (CSF) α-syn was considered to be the most reliable biomarker for synucleinopathies, such as PD (Atik et al., 2016), and DLB (Zhang et al., 2022). The total α-syn level was considered to be low in the CSF of PD patients and DLB patients. Additionally, α-syn has also been shown to be associated with the pathophysiology (Larson et al., 2012) and core CSF biomarkers (Monge-García et al., 2021) of Alzheimer’s disease (AD).

Previous studies demonstrated that air pollution can induce oxidative stress and neuroinflammation, both of which are implicated in the pathogenesis of synucleinopathies (Takahashi et al., 2007). Meanwhile, air pollution constituents may directly reach the brain via the olfactory system or the blood–brain barrier, potentially affecting α-synuclein aggregation and clearance (Doty, 2017). A post-mortem survey has shown that metal-rich nanoparticles which are associated with PM_2.5_ may be a novel pathway for synucleinopathies and Alzheimer’s disease (AD) pathogenesis (Calderón-Garcidueñas et al., 2020). Multiple systematic reviews found air pollution exposure might be associated with the onset of synucleinopathies, such as PD and MSA (Cristaldi et al., 2022). Recently, a large number of literature has evolved around the question of whether air pollution increases the risk of PD and other central nervous system (CNS) diseases (Block and Calderón-Garcidueñas, 2009). A study by Calderón-Garcidueñas et al. (2010) showed that exposure to highly polluted environments often leads to the accumulation of α-syn in the brain and immune response to α-syn. However, there were few comprehensive studies into the relationship between air pollution and α-syn in CSF. Therefore, the purpose of this study is to explore the influence of ambient air pollution on CSF α-syn levels among healthy urban older adults and to investigate the above effects among different genders, ages, seasons, and a history of coronary heart disease (CHD) groups.

## Materials and methods

### The CABLE database

The cross-sectional study consisted of participants from the Chinese Alzheimer’s Biomarker and LifestylE (CABLE) database (Hu et al., 2022). CABLE is an ongoing large independent cohort that mainly focuses on biomarkers and risk factors for neurodegenerative disorders, which was initiated in 2017. The database collected data on various biomarkers, including total α-syn levels in CSF. All of the participants were recruited from Qingdao Municipal Hospital, Shandong Province, China. All eligible participants were Han Chinese, with an age range of 40 to 90 years old. Individuals with major neurological disorders (e.g., head trauma, multiple sclerosis, central nervous system infection, epilepsy), major psychological disorders, malignant tumors, and genetic disorders were excluded. Each participant underwent a systematic neuropsychological, psychiatric, psychosocial, and clinical evaluation, blood and CSF sample collection. Their demographics and medical history data were obtained from a structured comprehensive questionnaire and an electronic record system. The above assessments and tests were all performed by specially trained neurologists. The CABLE database was approved by the ethics committee of Qingdao Municipal Hospital. All the participants or their representatives provided written informed consent following the Declaration of Helsinki.

### Study participants

Our study enrolled 933 healthy urban participants after excluding individuals with severe cognitive impairment. The general cognitive function of the participants in our study was evaluated using the Chinese Modified Mini-Mental State Examination (CM-MMSE), ranging from 0 to 30. The higher CM-MMSE scores indicate better cognitive ability. Participants who met the following criteria are considered cognitively normal: CM-MMSE scores >17 for illiterate, CM-MMSE scores >20 for those with 1–6 years of education, and CM-MMSE scores >24 for those with more than 6 years of education. Participants’ information regarding demographic characteristics, lifestyle, socioeconomic factors, and comorbidities was obtained from a standardized self-report questionnaire.

### Assessment of covariates

To mitigate the impact of individual differences among participants, the study adjusted for potential confounding factors, including age (continuous), gender (female or male), educational years (continuous), CM-MMSE (continuous), and BMI (continuous). Adjustment for lifestyle factors is as follows: cigarette use (yes or no), alcohol use (yes or no), and physical activity (yes or no). Adjustment for socioeconomic factors includes employment status (yes or no). Adjustment for comorbidities is as follows: hypertension (yes or no), diabetes mellitus (yes or no), and hyperlipidemia (yes or no).

### Ambient air pollution exposure assessment

In the CABLE study, the annual average concentration of particulate matter with aerodynamic diameter < 2.5 μm (PM_2.5_) was estimated from a national exposure assessment model based on random forest modeling combined with satellite aerosol optical depth (AOD), population visibility, meteorological data, and land use parameters (Meng et al., 2021). Overall, for the areas lacking ground-based PM_2.5_ monitoring stations, we applied two complementary models to predict and calculate PM_2.5_ concentration, including one with AOD and another without AOD. AOD models can be loosely described as prediction of air pollution concentration under 1 km × 1 km resolution using satellite data combined with a random forest model. In addition, our AOD model incorporated a range of variables as predictors, including land use parameters, population density, visibility, etc. When AOD information is missing, the non-AOD model will fill this gap to achieve full information coverage. The annual cross-validation R^2^ values ranged from 0.89 to 0.92. Mean annual ozone (O_3_) (Shaddick et al., 2018) concentrations and nitrogen dioxide (NO_2_) concentrations (Larkin et al., 2017) were obtained from the exposure products of Global Burden of Disease 2019. Specific details and methods can be seen in the Supplementary material. Finally, we assessed the air pollution exposure in the CABLE study based on the residential addresses of the participants. We adopted the 5-year average air pollution exposure concentrations since the establishment of CABLE as the independent variable.

### Cerebrospinal fluid α-syn

CSF was obtained via lumbar puncture. After the CSF was extracted, it was transported to the local laboratory in 10 mL polypropylene tubes within 2 h. The extract was centrifuged for 10 min at 2000 × g and was promptly snap-frozen in a-80 refrigerator for the next step of testing. The levels of CSF α-syn were measured by the enzyme-linked immunosorbent assay (ELISA) kit (Legend Max™ Human a-Synuclein, BioLegend Corporation) on the EnSpire Multimode Plate Reader (PerkinElmer Corporation). To maintain the stability of biomarkers in CSF, the thaw-freezing cycle was controlled within 2 cycles (del Campo et al., 2012). All CSF samples were repeatedly measured by experienced laboratory technicians who were blinded to clinical information about the participants. To minimize pre-analytical and analytical variability, we controlled the coefficient of variation of the α-syn to be lower than 20%.

### Data analysis

For the differences in groups, we used the Mann–Whitney U-test for continuous variables. To provide a visual representation of the data distribution, we used histograms to show the distribution of CSF α-synuclein levels (Supplementary Figure S1). Multivariate linear regression was conducted to investigate the linear relationship between air pollution and the CSF level of α-syn, using the concentration of ambient air pollution (including PM_2.5_, O_3_, and NO_2_) as the independent variable and CSF α-syn level as the dependent variable. Data on CSF α-syn level was transformed via the “car” package in R software. Eight individuals were excluded from the study due to their CSF total levels of α-syn > 7,000 pg/mL [which greatly exceeded the 95% confidence limit for the range of all CSF α-syn data (Mollenhauer et al., 2019)] to prevent the influence of extreme values on the results.

To increase the robustness of our results, the following sensitivity analyses were performed: 1) the analysis of the associations between air pollution indicators and CSF level of α-syn, with the air pollution indicators as dichotomous variables [dichotomized to the top quartile and the bottom 3 quartiles (Reuben et al., 2021)]; 2) the repeated analysis of the relationship between air pollution and CSF level of α-syn in 868 cognitively normal people; 3) the sensitivity analysis including the following four model (model 1–4). Model 1 did not adjust for any covariates; Model 2 only adjusts for age and gender; Model 3 included model 2 plus years of education and BMI; Model 4 included model 3 plus lifestyles (smoking, drinking habit, and physical activity), socioeconomic factor (employment), and comorbidities (hypertension, diabetes mellitus, hyperlipemia). Moreover, we performed subgroup analyses stratified by the factors that may be related to air pollution and CSF level of α-syn, such as age (mid-life vs. late-life), sex (female vs. male), the season for extracting CSF samples (cool vs. warm), and previous history of CHD (yes vs. no). The cool season was defined as the period from October to March, and the warm season was from April to September according to the classification used by Lee et al. (2017). Considering the complex coexistence of various components in air pollutants, we added a 2-way interaction to explore whether PM_2.5_, O_3,_ and NO_2_ have any interaction on CSF a-syn levels.

In addition, we used restricted cubic spline models (RCS) to flexibly model potential non-linear relationships and to explore the dose–response association between the CSF level of α-syn and air pollution. Due to our large sample size, 5 knots (5th, 25th, 50th, 75th, and 95th percentiles) were applied to the RCS model. The RCS model also adjusted for the most basic confounding factors, including age and gender. The inflection point for the CSF α-syn normalized score was estimated from a two-line piecewise linear model (Bhaskaran et al., 2018).

The R version 3.5.1 software (R Foundation for Statistical Computing) and SPSS 26.0 were used for data analysis and image rendering. For all the statistical tests, a two-tailed *p* < 0.05 was considered significant.^1^

## Results

### Participant characteristics

The mean age of the 933 urban participants was 62.57 ± 10.62 years and 40% were women. In all the participants, the average 5-year exposure concentrations were 53.53 μg/m^3^, 39.87 ppb, and 13.84 ppb for PM_2.5_, NO_2,_ and O_3_, respectively. The mean CSF level of α-syn was 1305.89 pg/mL (SD = 689.11 pg/mL) in the urban participants. Besides, the mean MMSE score was 27.77, Other details are presented in Table 1.

**Table 1:** Basic characteristics of included participants.

Variable	Total
N	933
Age, year, mean [SD]	62.57 [10.62]
Gender, female/male	363/569
Education, year, mean [SD]	10.80 [3.84]
CM-MMSE mean [SD]	27.77[2.42]
BMI, kg/m^2^, mean [SD]	25.49 [3.98]
Cigarette use, yes (%)	288 (30.87)
Alcohol use, yes (%)	281 (30.12)
Employment, yes (%)	310 (33.23)
Regular exercise, yes (%)	433 (46.41)
Hypertension, yes (%)	353 (37.86)
Diabetes mellitus, yes (%)	152 (16.31)
Hyperlipemia, yes (%)	35 (3.76)
Coronary heart disease, yes (%)	139 (14.91)
Stroke, yes (%)	34 (3.65)
Hamilton Depression Rating Scale, mean [SD]	0.48 (1.47)
Hamilton Anxiety Rating Scale, mean [SD]	0.67 (2.11)
Season, warm (April–September, %)	406 (43.88)
CSF total α-syn, pg/ml, mean [SD]	1305.46 [689.11]
Pollutant exposures, mean [SD]	
PM_2.5_, μg/m^3^	53.53 [6.20]
O_3_, ppb	39.87 [2.66]
NO_2_, ppb	13.84 [3.65]

SD, standardized deviation; CM-MMSE, China-Modified Mini Mental State Examination; BMI, body mass index (calculated as weight in kilograms divided by square of height in meters); NO_2_, nitrogen dioxide; O_3_, ozone; PM_2.5_, particulate matter with aerodynamic diameters less than 2.5 μm; ppb, parts per billion; CSF Cerebrospinal fluid; α-syn, alpha-synuclein.

### Difference between groups

Firstly, when air pollution indicators were dichotomized at the top quartile of exposure, we found group differences between the high PM_2.5_ group and the low PM_2.5_ group (*p* = 0.010) and between the high O_3_ group and the low O_3_ group (*p* = 0.041) in the urban participants. More details can be seen in Supplementary Figure S2.

### Associations between air pollution exposures and CSF α-syn levels

**Figure 1:**
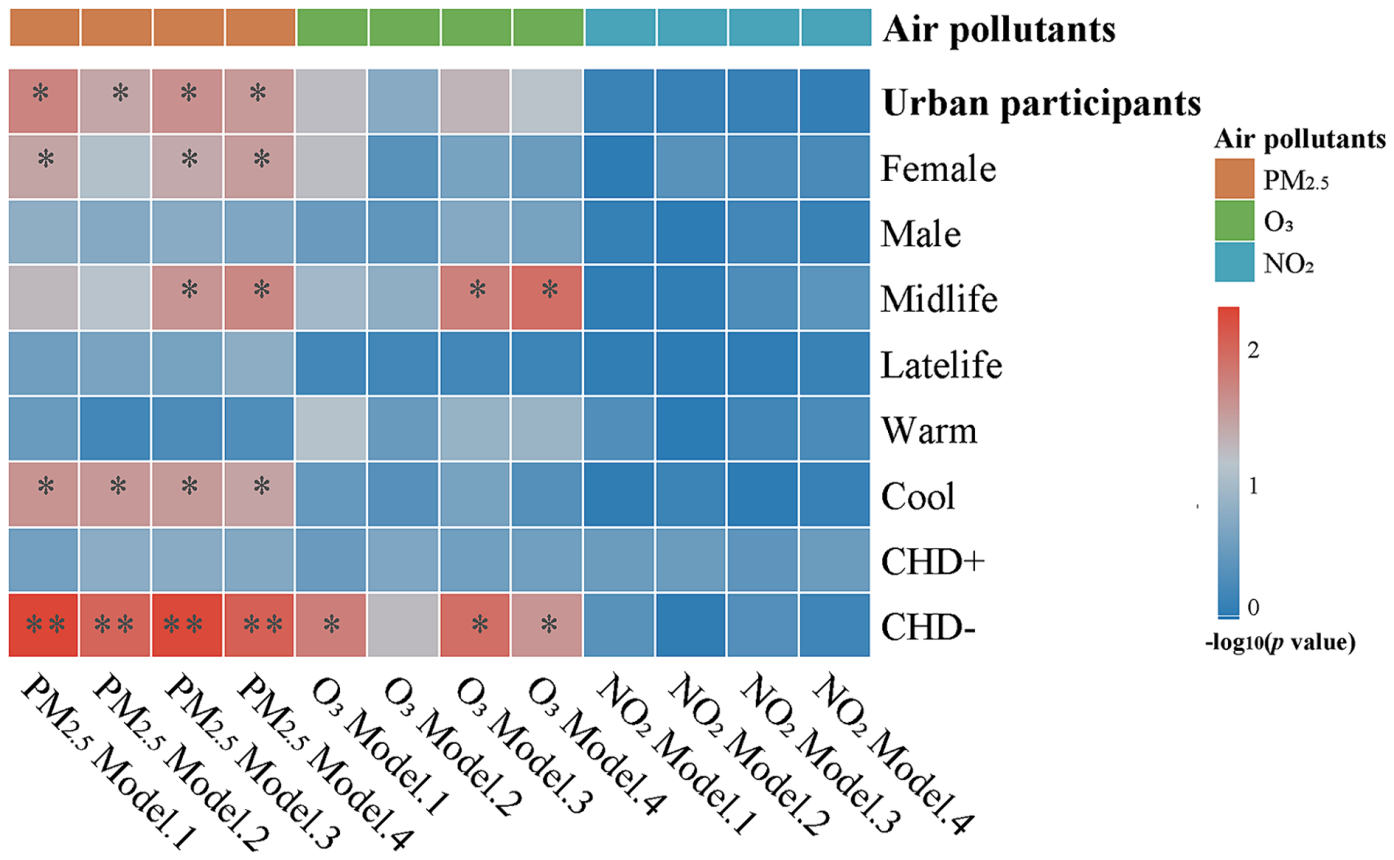
Linear associations of air pollution with CSF α-syn levels. A higher PM_2.5_ normalized score is associated with lower CSF a-syn levels in urban participants. These associations were more obvious in females, midlife, participants without CHD, or the cold season subgroup. The correlation for O_3_ was more significant in midlife or participants without CHD. Multiple linear regression models were used to examine the associations of air pollution with CSF α-syn levels. Model 1: non-adjustment; Model 2: adjusted for age and sex; Model 3: adjusted for model 2 + years of education and BMI; Model 4: adjusted for model 3 + lifestyles (smoking, drinking habit, and physical activity) + socioeconomic factor (employment) + comorbidities (hypertension, diabetes mellitus, hyperlipemia). Abbreviations: CSF, cerebrospinal fluid; α-syn, alpha-synuclein; PM_2.5_, particulate matter with aerodynamic diameters less than 2.5 μm; O_3_, ozone; NO_2_, nitrogen dioxide; CHD+, participants with coronary heart disease; CHD-, participants without coronary heart disease; * *p* < 0.05; ** *p* < 0.01.

As shown in Supplementary Table S1 and Figure 1, the results of multiple linear regression analysis were as follows. 1) An association between PM_2.5_ and CSF α-syn levels was observed in the urban participant (fully adjusted *p* = 0.029). Specifically, individuals exposed to higher levels of PM_2.5_ tended to have lower total α-syn levels in CSF. 2) Notably, the annual average concentration of O_3_ showed a borderline negative correlation with CSF α-syn levels in the urban participant (fully adjusted *p* = 0.067). 3) the association between NO_2_ and CSF α-syn levels was no longer statistically significant in the urban participants in all the models. No significant interactions were found between PM_2.5_, O_3_, and NO_2_, more details can be seen in Supplementary Table S2.

### Non-linear relationships between air pollution exposures and CSF α-syn levels

**Figure 2:**
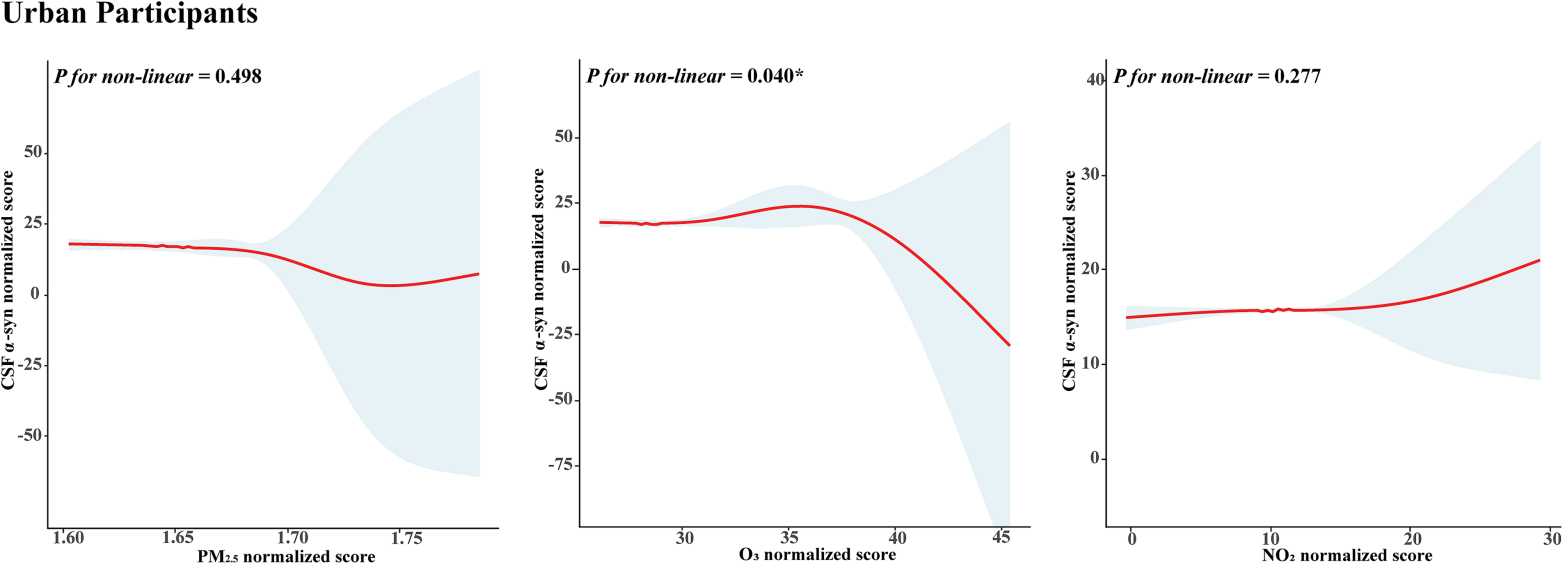
Non-linear association between air pollution and CSF α-syn level in urban participants. A non-linear association between O_3_ and CSF α-syn level was found after adjustment for age, and gender in urban participants. No significant non-linear correlation between PM_2.5_ and NO_2_ and CSF α-syn level was found. As O_3_ concentrations increase, the effect of O_3_ on CSF a-syn levels was small at the beginning, while CSF a-syn levels decreased rapidly at higher O_3_ concentrations. The blue area represents 95% of CIs. Abbreviations: CSF, cerebrospinal fluid; α-syn, alpha-synuclein; O_3_, ozone; PM_2.5_, particulate matter with aerodynamic diameters less than 2.5 μm; NO_2_, nitrogen dioxide; CIs, confidence interval; RCS: restricted cubic spline.

In Figure 2, we used the RCS to flexibly model and visualize the association of air pollution with CSF α-syn levels. For O_3_, the linear analysis did not show a significant statistical correlation, but the nonlinear analysis showed a nonlinear association between O_3_ and CSF α-syn levels after adjusting for sex and gender (*p* for nonlinearity = 0.040). According to the estimation from piecewise linear models, the peak for CSF α-syn levels among urban participants was at an O_3_ concentration of 37.9 ppb. With the increase of O_3_ concentration, the CSF of α-syn levels slightly decreased at the beginning, and then rapidly decreased at high concentrations of O_3_. To be specific, when the concentration of O_3_ was higher than 37.9 ppb, there was an obvious negative correlation.

### Subgroup analyses of healthy participants from urban areas

Subgroup analyses were conducted for urban residents according to gender, age, season, and the history of CHD. Our results revealed that females, those younger than 65 years, those who underwent CSF sampling during cold seasons, and those without a history of CHD were more susceptible to air pollution exposures. To be specific, in the urban participants, the concentration of PM_2.5_ was found to be negatively associated with CSF total level of α-syn in females (*β* = −0.116, *p* = 0.030), mid-aged adults (*β* = −0.105, *p* = 0.020), cold season subgroup (*β* = −0.092, *p* = 0.035), and the subgroup of no-CHD history (*β* = −0.095, *p* = 0.009); the concentration of O_3_ was found to be negatively associated with CSF total level of α-syn in mid-aged adults subgroup (*β* = −0.118, *p* = 0.012) and the subgroup of no-CHD history (*β* = −0.084, *p* = 0.026); and no association between NO_2_ and CSF α-syn levels were observed in any of these subgroups. More details can be seen in Supplementary Table S3. Scatterplots were presented in Supplementary Figure S3.

### Sensitivity analyses

We conducted multiple sensitivity analyses: 1) when air pollution indicators were dichotomized at the top quartile of exposure, the associations of PM_2.5_ with CSF α-syn levels remained statistically significant in the urban participant (fully adjusted *p* = 0.013). More details can be seen in Supplementary Table S4 and Supplementary Figure S4. 2) In the cognitively normal urban participant, PM_2.5_ still had a negative linear correlation with CSF α-syn levels (Supplementary Table S5; Supplementary Figure S5). 3) In addition, after adjusting for demographic characteristics, lifestyles, socioeconomic factors, and comorbidities, the results of the multivariable models were consistent with the unadjusted models.

## Discussion

This study found that exposure to PM_2.5_ might lead to a reduction in CSF levels of α-syn in urban participants. Moreover, the associations were more significant in females, mid-aged adults, participants without a CHD history, and the cold season subgroup. A non-linear relationship between O_3_ concentration and CSF α-syn levels was found. When the O_3_ concentration exceeded 37.9 ppb, CSF α-syn levels declined markedly. In summary, our analysis provided insights into possible mechanisms by which inhalation of air pollutants led to synucleinopathies.

The main results in our analyses were consistent with most previous animal and human autopsy studies showing an association between air pollution and α-syn accumulation in the brain. For example, a recent study demonstrated that PM_2.5_ exposure promoted α-syn pathology using α-syn A53T mice (Yuan et al., 2022). A recent study found that young individuals exposed to PM_2.5_, combustion, and friction ultrafine particulate matter, and industrial nanoparticles exhibited early neuropathological features of AD, PD (positive α-syn), frontotemporal lobar degeneration, and amyotrophic lateral sclerosis (Calderón-Garcidueñas et al., 2023). Another study found that exposure to O_3_ increased the misfolding of α-syn and induced a loss of inflammation response regulation (Valdés-Fuentes et al., 2024). Compared with these prior studies, our study had the advantage of more readily available CSF samples to detect α-syn. Besides, misfolded α-syn is a pathological feature of PD, and CSF α-syn is a highly sensitive biomarker for PD (Atik et al., 2016). In the brain, the sequestration of α-syn into fibrillar aggregates may be responsible for the changes in CSF α-syn levels. Most previous studies show that exposure to PM_2.5_ and O_3_ was linked to an elevated risk of PD (Cristaldi et al., 2022), and AD (Hussain et al., 2023). However, A retrospective cohort study in Korea found no association between O_3_ concentration and PD risk (Jo et al., 2021), in which the measurement error in exposure estimates tended to cause a bias toward a null.

Air pollution may decrease CSF α-syn levels in the following three ways. Firstly, exposure to air pollutants can have a direct impact on CSF α-syn levels. Yuan et al. found that exposure to PM_2.5_ directly accelerated α-syn aggregation and thus decreased the CSF α-syn levels (Yuan et al., 2022). Secondly, the most widely accepted possible mechanism is oxidative and inflammatory responses in the central nervous system (CNS). It has been shown that air pollutants could cause neuroinflammation and oxidative stress by directly entering the CNS of animals through the blood–brain barrier (BBB), subsequently leading to lower α-syn levels in the CSF (Takahashi et al., 2007; Fink, 2006). Another study indicated that environmental O_3_ generates reactive oxygen species (ROS) in the body. These ROS activated transcription factors such as the NF-κB factor, leading to oxidative stress and associated misfolding of α-synuclein protein (Valdés-Fuentes et al., 2024). Thirdly, air pollution may affect α-syn pathology through the olfactory bulb (OBs). OB has been reported to be one of the earliest regions affected by Lewy bodies (LBs), of which fibrotic α-syn is an important component (Doty, 2017). Air pollution may affect α-syn deposition in the olfactory bulb at an early stage and lead to dysosmia in early PD. Interestingly, recent research has found that nano-particulate matter and α-syn work synergistically to downregulate the expression of glutamate receptor A1 in the OBs and cortex, thereby affecting olfactory and memory functions in humans (Zhang et al., 2023).

In our study, we found a linear relationship that nearly reached statistical significance and a significant nonlinear relationship in urban individuals between O_3_ and CSF α-syn levels, which were consistent with the existing literature. Recently, a Canadian population-based article has reported positive associations between long-term ozone exposure and mortality of synucleinopathies (PD and MSA) (Zhao et al., 2021). O_3_ may affect the human body through oxidative stress (Pereyra-Muñoz et al., 2006) and systemic inflammatory response (Mumaw et al., 2016), further increasing the risk of synucleinopathies. Notably, we found that CSF of α-syn levels rapidly decreased when the O_3_ concentration exceeded 37.9 ppb, which provided important implications for the control of O_3_ concentration in the prevention of synucleinopathies.

In the subgroup analysis by gender, the association of PM_2.5_ with CSF α-syn levels was only observed in females, which was consistent with a study by Liu et al. showing evidence for the associations of PM_10_ and PM_2.5_ with PD in female never smokers (Liu et al., 2016). This gender difference may be explained by the finding that females had higher gas sensitivity and higher airway responsiveness to oxidants. The age difference observed in our study could be explained by the finding of Zheng et al. that the level of α-syn in CSF increased with age in the control group (Hong et al., 2010). The seasonal effects of PM_2.5_ exposure have been demonstrated in several articles. On the one hand, the sources and chemical composition of air particles may vary from season to season (Yang et al., 2022). On the other hand, PM_2.5_ may have a seasonal dependence in causing neuronal apoptosis, reducing the postsynaptic density of synaptic structural proteins (PSD-95) and the expression of synaptic functional protein level n-methyl-d-aspartate (NMDA) receptor subunit (NR2B) (Chen et al., 2017). Our results revealed that the associations were more pronounced in individuals without a history of CHD. A possible reason might be that patients with CHD may be more likely to produce Ubiquitin carboxyl-terminal esterase L1 (UCHL1) due to myocardial damage, and UCHL1 plays a vital role in the deubiquitination and stabilization of α-synuclein (Wilkinson et al., 1989). However, since our study had a small sample size (only 139 urban residents with coronary heart disease), the conclusion should be drawn with caution.

Here are the strengths of our study. First, our study had a large sample size and adjusted for many potential confounding factors. Second, we further conducted subgroup analyses according to gender, age, season, and the history of CHD to assess whether these factors modify the association between air pollution and CSF α-syn levels. However, some limitations should be discussed. Firstly, our study was a cross-sectional observational study, which thus could not make a causal inference. In the future, longitudinal studies are warranted. Secondly, air pollution exposure estimates in our study were based on participants ‘home addresses but lacked mobility-related air pollution exposure data, so measurement error may have been present. Future research could consider the possibility of participants ‘migration or activity when we estimate the exposure to air pollution. Thirdly, the method for the determination of CSF α-syn levels by ELISA assay has the advantages of being quantitative, efficient, easy to analyze a large sample set, and highly sensitive (Majbour et al., 2022). In recent years, seeded-amplification assay (SAA) has gained increasing attention as a new method to detect α-syn aggregates in CSF. Future studies could combine the SAA and ELLSA techniques to measure the CSF α-syn levels (Majbour et al., 2022). Fourthly, unmeasured variables, such as genetic risk factors or dietary habits, may introduce residual confounding in the observational studies. Further research incorporating these variables is essential to better understand their potential impact. Fifthly, the use of the CABLE database might cause selection bias in the population. To avoid this, we only included healthy participants and conducted a sensitivity analysis on cognitively normal participants. Our results remained robust after conducting a sensitivity analysis. Future research needs to further verify the relationship in databases specifically targeting α-synuclein pathology.

## Conclusion

Exposure to air pollution, especially PM_2.5,_ and O_3_, was associated with reduced CSF α-synuclein levels in urban older participants. The association was more pronounced in females, mid-aged adults, participants without a CHD history, and the cold season subgroup. This discovery could deepen our understanding of the potential impact of air pollution on the nervous system. Tackling air pollution issues may facilitate the prevention of synucleinopathies and support policymakers in devising effective public health policies and individual protective strategies.

## Data Availability

The raw data supporting the conclusions of this article will be made available by the authors, without undue reservation.

## Footnotes

^1^ http://www.r-project.org/
