# Supplementary material for: Association between air pollution and cerebrospinal fluid alpha-synuclein in urban elders: the CABLE study
Source: Front Aging Neurosci. 2024 Aug 30;16:1422772. doi: 10.3389/fnagi.2024.1422772 (PMC11392785; doi:10.3389/fnagi.2024.1422772)
Supplement: Supplementary file 1 [file Data_Sheet_1.docx]

**Association between air pollution and cerebrospinal fluid alpha-synuclein in urban elders: the CABLE Study**

**Running title: Air pollution and cerebrospinal fluid alpha-synuclein**

**Content:**

**Supplementary Table S1 Associations between ambient air pollutants and CSF α-syn levels in non-demented urban participants……………………………………………………………………………………………..………………… 2**

**Supplementary Table S2: The result of interaction between PM_2.5_, O_3_, and NO_2_………………………………..…....3**

**Supplementary Table S3: Subgroup analyses of urban participants without dementia by age, gender, season, and a history of coronary heart disease…………………………………………………………………………………….....4**

**Supplementary Table S4: Associations between exposure to high/low ambient air pollutants and CFS α-syn levels in urban participants……………………………………………………………………………………………..………..7**

**Supplementary Table S5: Sensitivity analysis of** **the associations between ambient air pollutants and CFS α-syn levels in cognitively normal urban participants……………………………………………………………….…………8**

**Supplementary Figure S1: The histograms of CSF α-synuclein levels…………………………….…………….………9**

**Supplementary Figure S2: CSF α-syn levels in different air pollution groups…………………..……………………10**

**Supplementary Figure S3: Subgroup analyses of urban participants without dementia by age, gender, season and a history of coronary heart disease……………………………………………………………………………….……..13**

**Supplementary** **Figure S4: Associations between exposure to high/low ambient air pollutants and CFS α-syn levels in healthy participants………………………………………………………………………………………….…….......14**

**Supplementary Figure S4: Sensitivity analysis of** **the associations between ambient air pollutants and CFS α-syn levels in cognitively normal urban participants………………………………………………………………………...15**

**Supplementary method of exposure assessment…………………………………………………………………..........16**

**Supplementary Table S1 Associations between ambient air pollutants and CSF α-syn levels in non-demented urban participants**

| **Ambient**  **air pollutants** | **Model 1** | |  | **Model 2** | |  | **Model 3** | |  | **Model 4** | |
| --- | --- | --- | --- | --- | --- | --- | --- | --- | --- | --- | --- |
|  | **Beta** | ***P value*** |  | **Beta** | ***P value*** |  | **Beta** | ***P value*** |  | **Beta** | ***P value*** |
| **Urban participants** |  |  |  |  |  |  |  |  |  |  |  |
| PM_2.5_ | -0.0773 | **0.0182** |  | -0.0681 | **0.0369** |  | -0.0749 | **0.0238** |  | -0.0731 | **0.0285** |
| O_3_ | -0.0621 | 0.0581 |  | -0.0450 | 0.1759 |  | -0.0670 | 0.0501 |  | -0.0631 | 0.0673 |
| NO_2_ | 0.0112 | 0.7350 |  | -0.0100 | 0.7642 |  | 0.0097 | 0.7804 |  | 0.0076 | 0.8298 |

Standardized beta estimates are derived from multiple-variable linear models.

Model 1: non-adjustment;

Model 2: adjusted for age and sex;

Model 3: adjusted for model 2 + years of education and BMI;

Model 4: adjusted for model 3 + lifestyles (smoking, drinking habit, and physical activity) +socioeconomic factor (employment)+comorbidities (hypertension, diabetes mellitus, hyperlipemia);

Abbreviations: CFS, cerebrospinal fluid; α-syn, alpha-synuclein; NO_2_, nitrogen dioxide; PM_2.5_, particulate matter with aerodynamic diameters less than 2.5 µm; O_3_, ozone;

**Supplementary Table S2: The result of interaction between PM_2.5_, O_3_, and NO_2._**

| **Interaction** | **CFS α-syn** | |
| --- | --- | --- |
|  | **Beta** | ***P value*** |
| **Urban participants** |  |  |
| PM_2.5_ * O_3_ | 0.520 | 0.499 |
| PM_2.5_ * NO_2_ | 0.181 | 0.853 |
| O_3_ * NO_2_ | 0.005 | 0.498 |

The air pollutant concentrations for each participant were based on the 5-year average from 2013 to 2017.

All of the above interactions were adjusted for age, sex, years of education, BMI, smoking, drinking habit, physical activity, employment, hypertension, diabetes mellitus, and hyperlipemia.

Abbreviations: CFS, cerebrospinal fluid; α-syn, alpha-synuclein; NO_2_, nitrogen dioxide; PM_2.5_, particulate matter with aerodynamic diameters less than 2.5 µm; O_3_, ozone;

**Supplementary Table S3** **Subgroup analyses of urban participants without dementia by age, gender, season, and a history of coronary heart disease**

| **Group** | | | **CFS α-syn** | | | | | | | | | | |
| --- | --- | --- | --- | --- | --- | --- | --- | --- | --- | --- | --- | --- | --- |
|  |  |  | **Model 1** | |  | **Model 2** | |  | **Model 3** | |  | **Model 4** | |
|  |  |  | **Beta** | ***P value*** |  | **Beta** | ***P value*** |  | **Beta** | ***P value*** |  | **Beta** | ***P value*** |
| **PM_2.5_** |  |  |  |  |  |  |  |  |  |  |  |  |  |
|  | Gender | Female | -0.1106 | **0.0352** |  | -0.0898 | 0.0816 |  | -0.1092 | **0.0395** |  | -0.1163 | **0.0304** |
|  |  | Male | -0.0599 | 0.1540 |  | -0.0562 | 0.1830 |  | -0.0579 | 0.1732 |  | -0.0541 | 0.2078 |
|  | Age | < 65 | -0.0852 | 0.0527 |  | -0.0790 | 0.0730 |  | -0.1005 | **0.0256** |  | -0.1052 | **0.0198** |
|  |  | ≥ 65 | -0.0538 | 0.2740 |  | -0.0586 | 0.2289 |  | -0.0580 | 0.2361 |  | -0.0699 | 0.1587 |
|  | Season | Warm | -0.0505 | 0.3100 |  | -0.0239 | 0.6309 |  | -0.0338 | 0.5154 |  | -0.0355 | 0.5024 |
|  |  | Cool | -0.0974 | **0.0253** |  | -0.0956 | **0.0281** |  | -0.0947 | **0.0293** |  | -0.0921 | **0.0345** |
|  | CHD | Yes | 0.0968 | 0.2570 |  | 0.1199 | 0.1650 |  | 0.1194 | 0.1710 |  | 0.1193 | 0.1940 |
|  |  | No | -0.0999 | **0.0049** |  | -0.0917 | **0.0096** |  | -0.1002 | **0.0054** |  | -0.0952 | **0.0086** |
| **O3** | Gender | Female | -0.0992 | 0.0593 |  | -0.0428 | 0.4230 |  | -0.0655 | 0.2352 |  | -0.0587 | 0.2992 |
|  |  | Male | -0.0434 | 0.3020 |  | -0.0383 | 0.3700 |  | -0.0578 | 0.1861 |  | -0.0518 | 0.2409 |
|  | Age | < 65 | -0.0709 | 0.1070 |  | -0.0644 | 0.1550 |  | -0.1107 | **0.0172** |  | -0.1180 | **0.0117** |
|  |  | ≥ 65 | -0.0235 | 0.6330 |  | -0.0233 | 0.6327 |  | -0.0242 | 0.6296 |  | -0.0206 | 0.6837 |
|  | Season | Warm | -0.0882 | 0.0764 |  | -0.0514 | 0.3168 |  | -0.0798 | 0.1352 |  | -0.0825 | 0.1275 |
|  |  | Cool | -0.0423 | 0.3330 |  | -0.0348 | 0.4300 |  | -0.0536 | 0.2328 |  | -0.0335 | 0.4595 |
|  | CHD | Yes | 0.0882 | 0.3020 |  | 0.1084 | 0.2090 |  | 0.0974 | 0.2680 |  | 0.1015 | 0.2710 |
|  |  | No | -0.0852 | **0.0166** |  | -0.0428 | 0.4230 |  | -0.0655 | 0.2352 |  | -0.0837 | **0.0258** |
| **NO_2_** |  |  |  |  |  |  |  |  |  |  |  |  |  |
|  | Gender | Female | 0.0041 | 0.9380 |  | -0.0426 | 0.4210 |  | -0.0341 | 0.5431 |  | -0.0336 | 0.5598 |
|  |  | Male | 0.0103 | 0.8070 |  | 0.0038 | 0.9290 |  | 0.0222 | 0.6168 |  | 0.0147 | 0.7438 |
|  | Age | < 65 | 0.0079 | 0.8570 |  | -0.0081 | 0.8570 |  | 0.0313 | 0.5079 |  | 0.0405 | 0.3979 |
|  |  | ≥ 65 | -0.0090 | 0.8560 |  | -0.0052 | 0.9150 |  | -0.0059 | 0.9078 |  | -0.0169 | 0.7410 |
|  | Season | Warm | 0.0356 | 0.4750 |  | -0.0009 | 0.9849 |  | 0.0239 | 0.6528 |  | 0.0329 | 0.5460 |
|  |  | Cool | -0.0061 | 0.8890 |  | -0.0167 | 0.7070 |  | 0.0041 | 0.9300 |  | -0.0132 | 0.7776 |
|  | CHD | Yes | -0.0885 | 0.3010 |  | -0.0892 | 0.2980 |  | -0.0779 | 0.3720 |  | -0.0987 | 0.2970 |
|  |  | No | 0.0288 | 0.4210 |  | 0.0054 | 0.8807 |  | 0.0276 | 0.4698 |  | 0.0172 | 0.6553 |

Standardized beta estimates are derived from multiple-variable linear models.

Bold indicated that the results were statistically significant.

Pollutant exposure was estimated using averaged 5-year exposure.

Warm season: April to September; Cool season: October to March;

Model 1: non-adjustment;

Model 2: adjusted for age and sex;

Model 3: adjusted for model 2 + years of education and BMI;

Model 4: adjusted for model 3 + lifestyles (smoking, drinking habit, and physical activity) +socioeconomic factor (employment)+comorbidities (hypertension, diabetes mellitus, hyperlipemia);

Abbreviations: CFS, cerebrospinal fluid; α-syn, alpha-synuclein; NO_2_, nitrogen dioxide; O_3_, ozone; CHD, coronary heart disease; PM_2.5_, particulate matter with aerodynamic diameters less than 2.5 µm;

**Supplementary Table S4: Associations between exposure to high/low ambient air pollutants and CFS α-syn levels in urban participants**

| **Ambient air pollutants** | | **Model 1** | | **Model 2** | | **Model 3** | | **Model 4** | |
| --- | --- | --- | --- | --- | --- | --- | --- | --- | --- |
|  |  | **Beta** | ***P value*** | **Beta** | ***P value*** | **Beta** | ***P value*** | **Beta** | ***P value*** |
| **Urban participants** | |  | |  | |  | |  | |
|  | PM_2.5_ | -0.2073 | **0.0060** | -0.1765 | **0.0196** | -0.1898 | **0.0134** | -0.1916 | **0.0132** |
|  | O_3_ | -0.1248 | 0.0992 | -0.0799 | 0.3001 | -0.1357 | 0.0897 | -0.1227 | 0.1288 |
|  | NO_2_ | 0.0288 | 0.6970 | 0.0051 | 0.9446 | 0.0251 | 0.7406 | 0.0237 | 0.7571 |

Considering exposure as a binary variable, linear associations of ambient air pollutants and alpha-synuclein in CFS. The top quarter of air exposure was regarded as high concentration air exposure. The bottom 3 quartiles of air pollutant exposure were regarded as low concentration air exposure.

Standardized beta estimates are derived from multiple-variable linear models.

Bold indicated that the results were statistically significant.

Pollutant exposure was estimated using averaged 5-year exposure.

Model 1: non-adjustment; Model 2: adjusted for age and sex; Model 3: adjusted for model 2 + years of education and BMI; Model 4: adjusted for model 3 + lifestyles (smoking, drinking habit, and physical activity) +socioeconomic factor (employment)+comorbidities (hypertension, diabetes mellitus, hyperlipemia);

Abbreviations: CFS, cerebrospinal fluid; α-syn, alpha-synuclein; NO_2_, nitrogen dioxide; PM_2.5_, particulate matter with aerodynamic diameters less than 2.5μm; O_3_, ozone;

**Supplementary Table S5:** **Sensitivity analysis of** **the associations between ambient air pollutants and CFS α-syn levels in cognitively normal** **urban participants**

| **Ambient air pollutants** | | **Model 1** | | **Model 2** | | **Model 3** | | **Model 4** | |
| --- | --- | --- | --- | --- | --- | --- | --- | --- | --- |
|  |  | **Beta** | ***P value*** | **Beta** | ***P value*** | **Beta** | ***P value*** | **Beta** | ***P value*** |
| **Urban participants** | |  |  |  |  |  |  |  |  |
|  | PM_2.5_ | -0.0830 | **0.0145** | -0.0749 | **0.0272** | -0.0832 | **0.0160** | -0.0801 | **0.0210** |
|  | O_3_ | -0.0584 | 0.0862 | -0.0446 | 0.1965 | -0.0670 | 0.0595 | -0.0619 | 0.0835 |
|  | NO_2_ | 0.0144 | 0.6730 | -0.0047 | 0.8917 | 0.0151 | 0.6763 | 0.0091 | 0.8031 |

Standardized beta estimates are derived from multiple-variable linear models.

Bold indicated that the results were statistically significant.

Pollutant exposure was estimated using averaged 5-year exposure.

Model 1: non-adjustment;

Model 2: adjusted for age and sex;

Model 3: adjusted for model 2 + years of education and BMI;

Model 4: adjusted for model 3 + lifestyles (smoking, drinking habit, and physical activity) +socioeconomic factor (employment)+comorbidities (hypertension, diabetes mellitus, hyperlipemia);

Abbreviations: CFS, cerebrospinal fluid; α-syn, alpha-synuclein; NO_2_, nitrogen dioxide; PM_2.5_, particulate matter with aerodynamic diameters less than 2.5μm; O_3_, ozone;

**Supplementary Figure S1: The histograms of CSF α-synuclein levels**

**
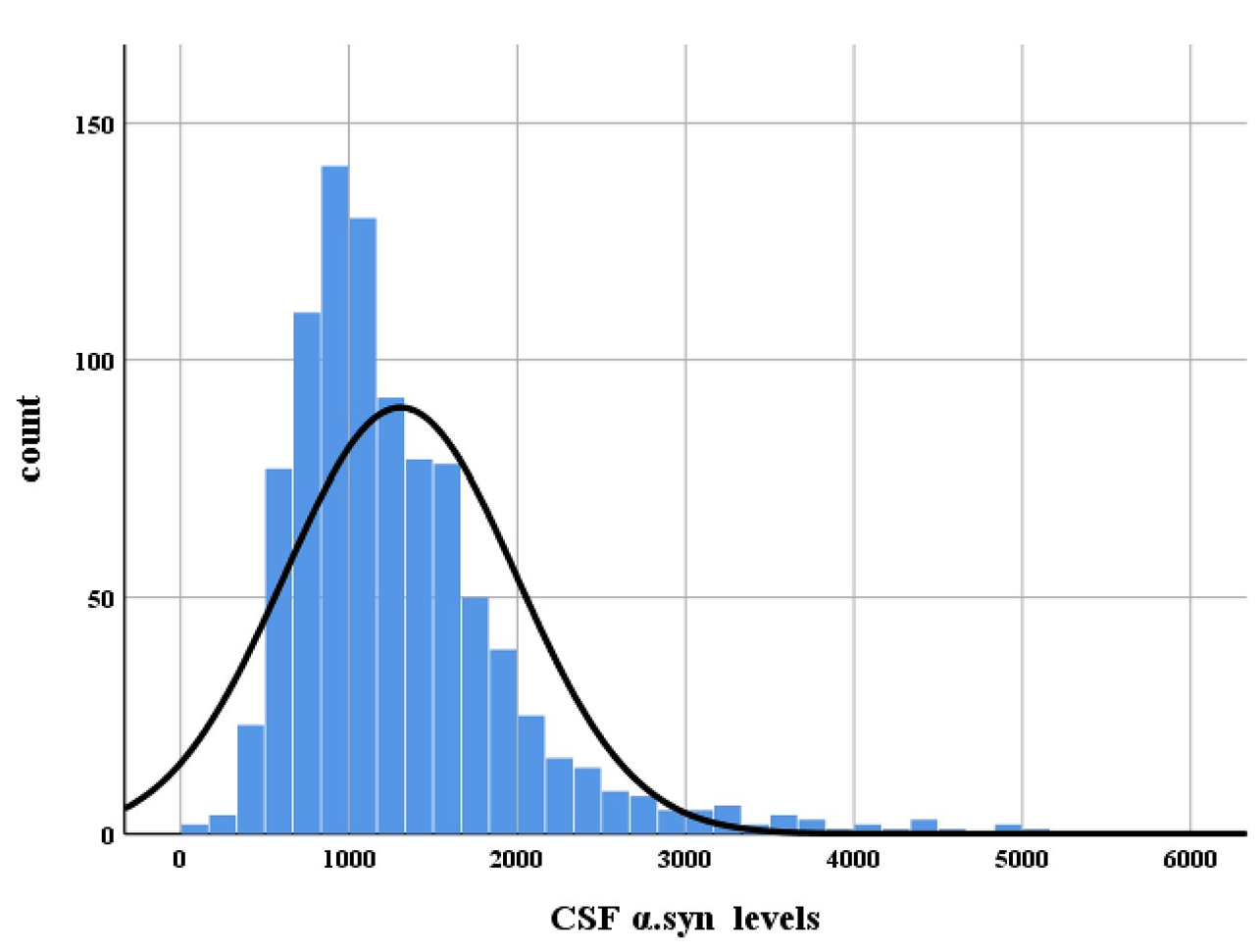
**

**Supplementary Figure S2: CSF α-syn levels in different air pollution groups.**

**
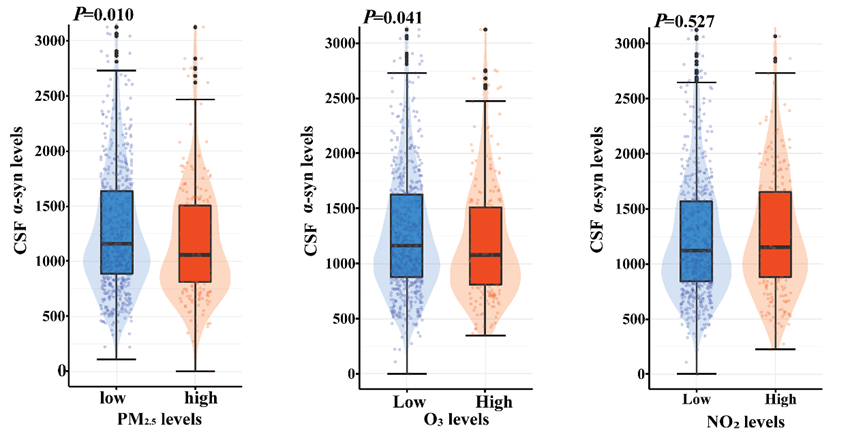
**

The CSF α-syn levels were lower in the high PM_2.5_ group (A) and high O_3_ group (B). There was no obviously group difference between high NO_2_ group and low NO_2_ group (C). P-values were assessed by Mann-Whitney U test. The top quarter of air exposure was regarded as high-concentration air exposure. The bottom 3 quartiles of air pollutant exposure were regarded as low-concentration air exposure.

Abbreviations: CSF, cerebrospinal fluid; α-syn, alpha-synuclein; PM_2.5_, particulate matter with aerodynamic diameters less than 2.5 µm; NO_2_, nitrogen dioxide; O_3_, ozone.

**Supplementary Figure S3: Subgroup analyses of urban participants without dementia by age, gender, season, and a history of coronary heart disease.**


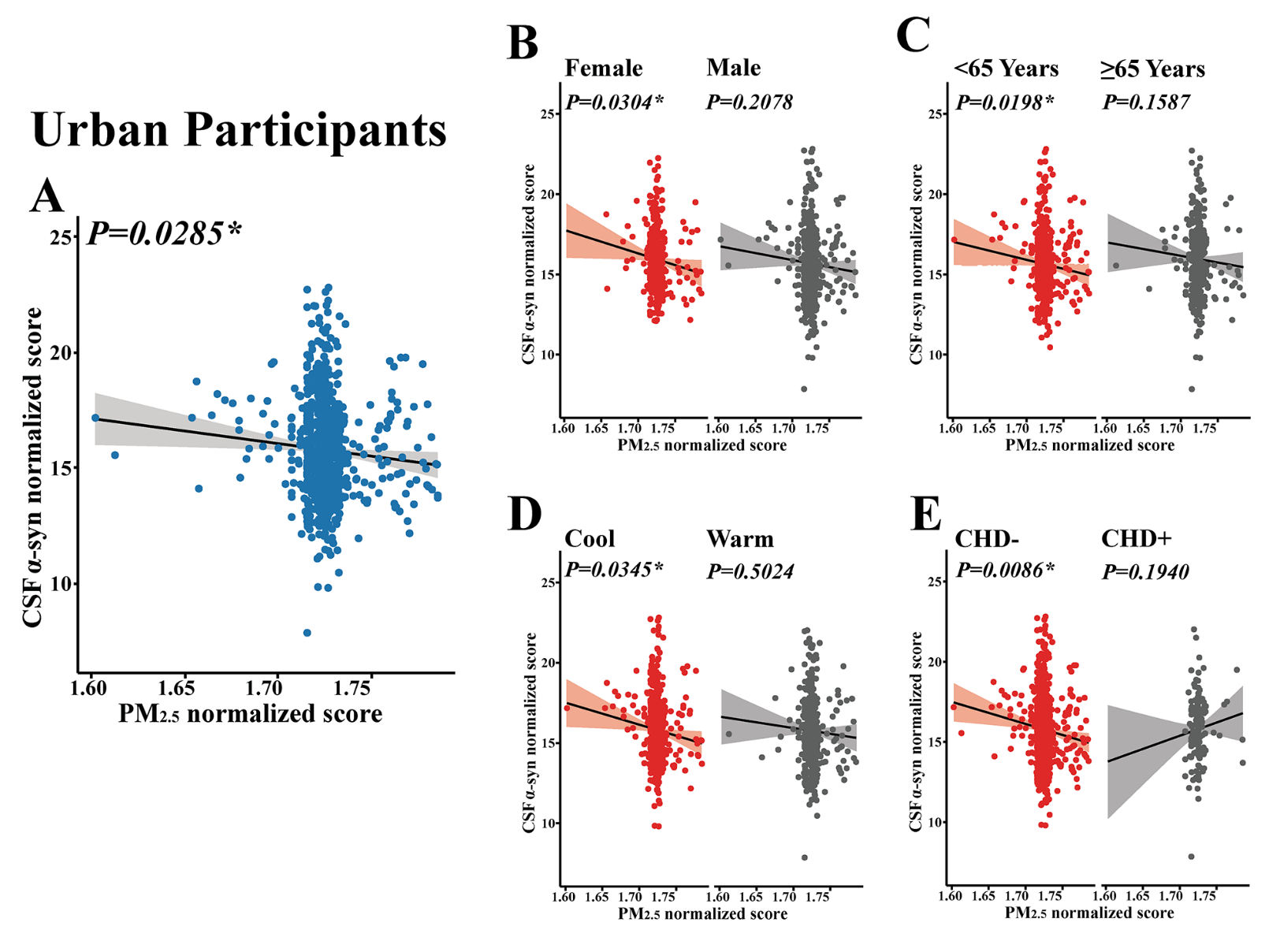
**PM_2.5_**

**O_3_**


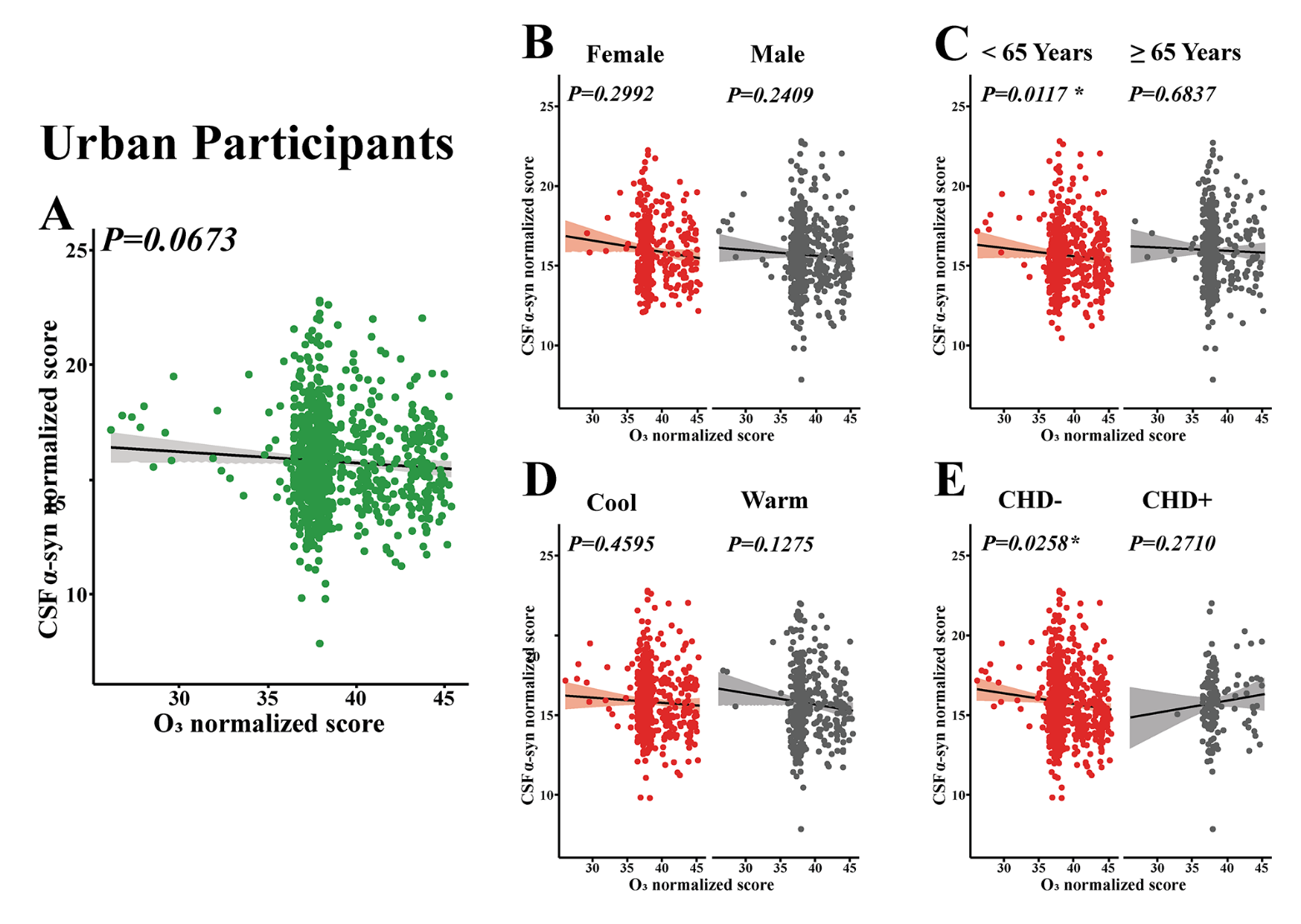


**
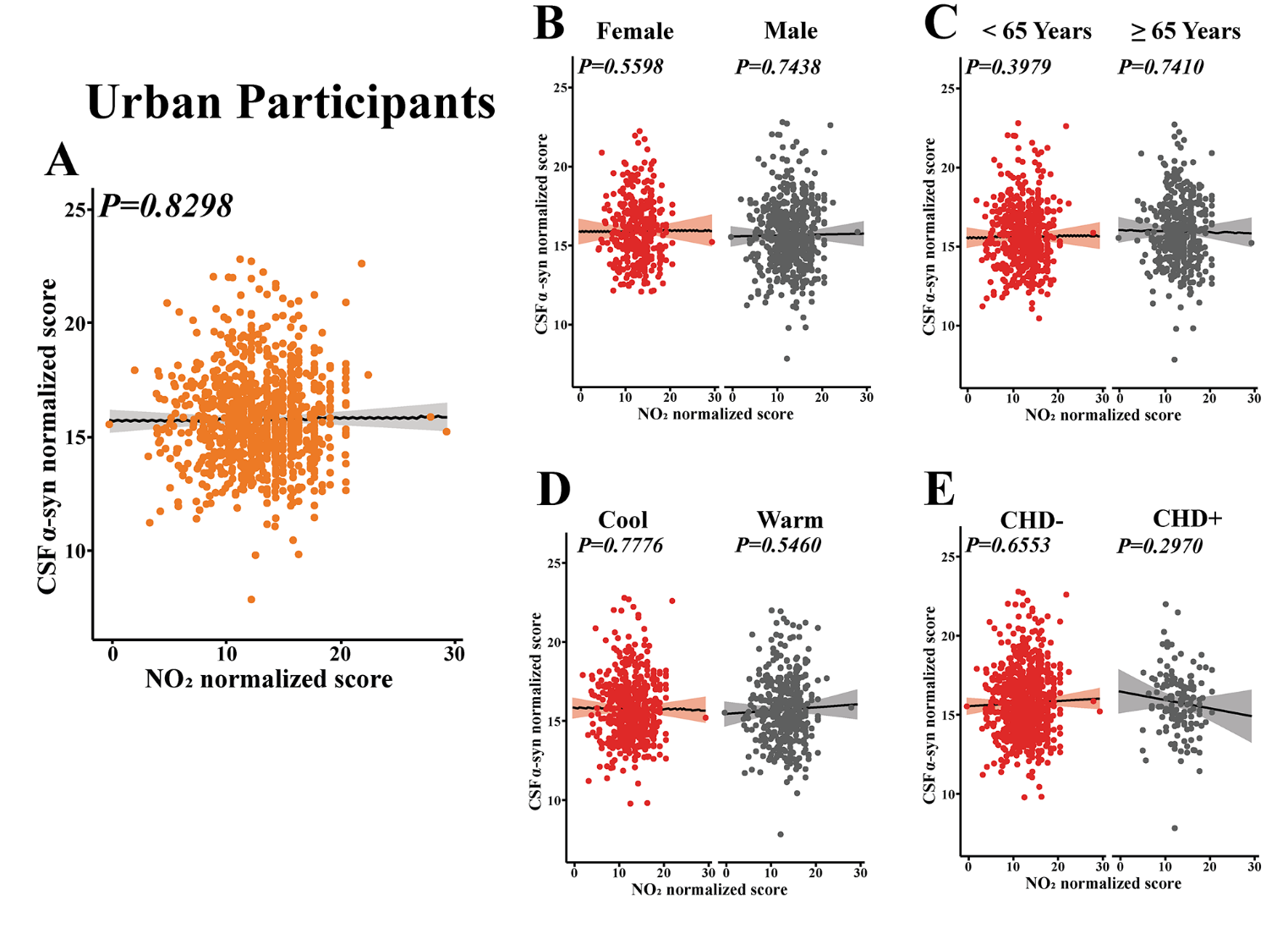
NO_2_**

**Supplementary Figure S4: Associations between exposure to high/low ambient air pollutants and CFS α-syn levels in healthy participants**

**
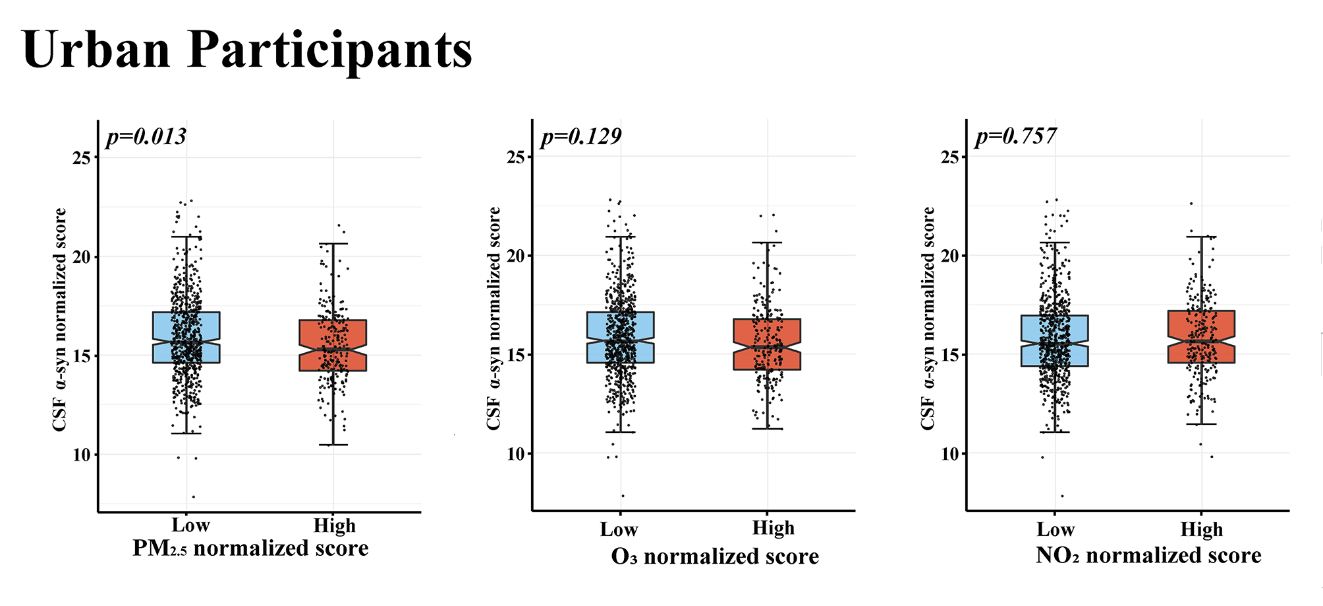
**

**Supplementary Figure S5: Sensitivity analysis of** **the associations between ambient air pollutants and CFS α-syn levels in cognitively normal urban participants**


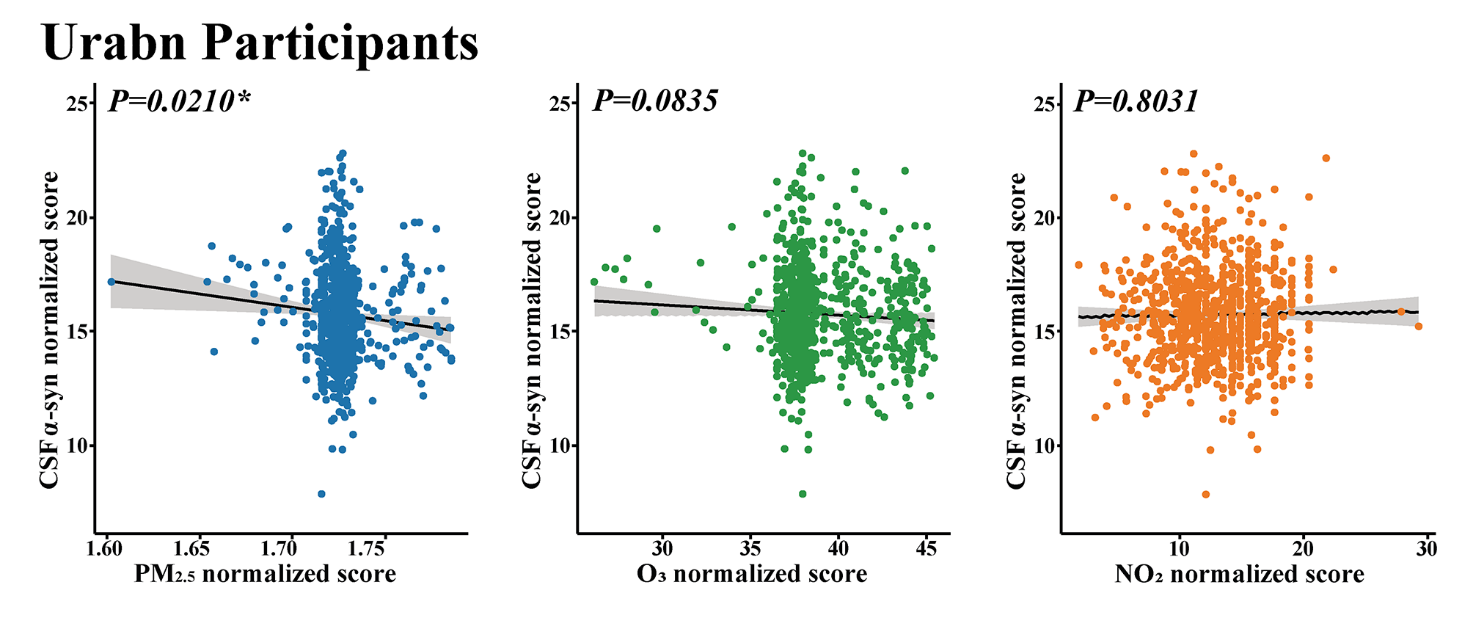


Abbreviations: CFS, cerebrospinal fluid; NO_2_, nitrogen dioxide; PM_2.5_, particulate matter with aerodynamic diameters less than 2.5μm; O_3_, ozone; CHD, coronary heart disease; RCS: Restricted cubic spline.

**Supplementary method of exposure assessment**

**PM_2.5_**

This study developed a national exposure assessment model using random forest modeling and combining satellite aerosol optical depths (AOD), meteorological data, land use parameters, population, and visibility data. To fill the gap in PM_2.5_ predictions caused by missing AOD at 1-km resolution, two models were built, one with AOD and one without AOD (Meng et al., 2021).

The China National Environmental Monitoring Center (CNEMC) provided ground measured hourly PM_2.5_ concentrations within the study domain from the China National Air Quality Monitoring Network at http://www.cnemc.cn/. In this study, we used the MAIAC (Multi-Angle Implementation of Atmospheric Correction) AOD data with a 1-km spatial resolution. The high resolution AOD was searched based on the MAIAC algorithm of the Moderate Resolution Imaging Spectroradiometer (MODIS) aboard the Terra and Aqua satellites. The gridded MAIAC AOD data was obtained from the Earth Data portal (https://search.earthdata.nasa.gov). The 2013-2019 AOD data was used for model development and the 2005-2012 data is used for forecasting. To integrate all parameters, we used a 1 km resolution modeling grid based on the MAIAC AOD. The simulated daily PM_2.5_ concentrations were measured based on the results of MERRA-2 (Modern-EraRestitute Analysis for Research and Applications, Version 2). Hourly visibility data from ground meteorological monitoring stations were obtained from National Oceanic and Atmospheric Administration (NOAA) of United States, and Cloud fraction (CF) data at 5-km resolution from Aqua and Terra (MYD06_L2 and MOD06_L2) were from the Earth Data portal (<https://search.earthdata.nasa.gov>).

The model for predicting PM_2.5_ levels was combined MAIAC AOD, MERRA-2 simulated PM_2.5_ concentrations, visibility, meteorological variables, land use data and population data. using a random forest model. We have built the random forest model with AOD on days and at grid cells of available AOD (AOD model) and without AOD ( non-AOD model ) on all days and at all grid cells, respectively. The non-AOD model contains the same forecast factors as the AOD model except for the MAIAC AOD.

**O_3_**

Ozone ground measurements were estimated using Bayesian maximum entropy combined with chemical transport models. Ozone monitoring data were collected from the Tropospheric Ozone Assessment Report (TOAR). Besides TOAR, our analysis applied ozone data from the China National Environmental Monitoring Center ( CNEMC ) network, which contained ground-level ozone measurements for China from 2013-2019. All of the observations were transformed to provide the six-month ozone season average of eight-hour daily maximum ozone concentrations. A combination of global atmospheric chemical transport models were applied, and many of these models simulate specific dynamics of the Chemistry-Climate Model Initiative (CCMI).In order to create a multi-model composite of the specified-dynamics models in each year from 1990 to 2017, we used the M3Fusion method.

**NO_2_**

Annual average surface NO_2_ concentration s at 1km × 1km resolution were estimated using a land-use regression model, combining inputs from the road network and other land-use variables, and satellite NO_2_ column observations from SCIAMACHY and GOME-2 (Geddes et al., 2016; Larkin et al., 2017). We aggregated NO_2_ concentrations from this dataset from its native 100m x 100m resolution globally to 1km x 1km. The final result used for estimating the global burden of disease from NO2 is a global, 0.0083 × 0.0083-degree (approximately 1km × 1km) resolution dataset of annual average surface NO_2_ concentrations from 1990 to 2019.(Ma et al., 2022)

**References:**

Geddes, J. A., Martin, R. V., Boys, B. L., & van Donkelaar, A. (2016). Long-Term Trends Worldwide in Ambient NO2 Concentrations Inferred from Satellite Observations. *Environ Health Perspect*, *124*(3), 281-289. <https://doi.org/10.1289/ehp.1409567>

Larkin, A., Geddes, J. A., Martin, R. V., Xiao, Q., Liu, Y., Marshall, J. D., Brauer, M., & Hystad, P. (2017). Global Land Use Regression Model for Nitrogen Dioxide Air Pollution. *Environ Sci Technol*, *51*(12), 6957-6964. <https://doi.org/10.1021/acs.est.7b01148>

Ma, Y. H., Chen, H. S., Liu, C., Feng, Q. S., Feng, L., Zhang, Y. R., Hu, H., Dong, Q., Tan, L., Kan, H. D., Zhang, C., Suckling, J., Zeng, Y., Chen, R. J., & Yu, J. T. (2022). Association of Long-term Exposure to Ambient Air Pollution With Cognitive Decline and Alzheimer's Disease-Related Amyloidosis. *Biol Psychiatry*. <https://doi.org/10.1016/j.biopsych.2022.05.017>

Meng, X., Liu, C., Zhang, L., Wang, W., Stowell, J., Kan, H., & Liu, Y. (2021). Estimating PM(2.5) concentrations in Northeastern China with full spatiotemporal coverage, 2005-2016. *Remote Sens Environ*, *253*. <https://doi.org/10.1016/j.rse.2020.112203>
